# An interview designed to promote mental health in organizations

**DOI:** 10.1192/j.eurpsy.2023.1899

**Published:** 2023-07-19

**Authors:** D. L. Peçanha

**Affiliations:** USE- School Health Unit, University Federal of San Carlos - UFSCar, Sao Paulo, Brazil

## Abstract

**Introduction:**

In a broad literature review on the subject we did not find structured interviews in the context of mental health in organizations. However, interviews are in common use in the business field. In Family Science assessment methods of families have fallen into two main categories. The first one is comprised of methods based on the evaluation of family members’ individual answers, while the second is based on the evaluation of group answers. The Structured Family Business Interview (SBFI) presented is based in important systemic studies and psychological practices with families and it belongs to the second method

**Objectives:**

The purposes of this study is present a structured interview called the Structured Family Business Interview (SBFI) that is a theoretical and practical contribution to access and to promote mental health in organizations.

**Methods:**

The Structured Family Business Interview (SFBI) is a structured interview comprised of six tasks which are assigned to the family as a group some of them are hypothetical or role-play type, and they are addressed to a particular family group in the family business.

The relational processes is observed first-hand by the interviewer and by a trained observer who audio-records and documents the non-verbal signals.

**Results:**

This technique was developed and tested in family firm context. In this section we will present illustrative answers to various dimensions studied in a large research project in mental health with family businesses. Results show good indicators of the SFBI capacity to assess dynamic and systemics dimensions of the teams in family firm. Those dimensions are: communication, rules, roles, conflicts, integration and aggressiveness analyzed to promote health resources and human and organizational development. The research with several work teams indicate that it allows for a precise evaluation of the variables.

**Conclusions:**

The Structured Family Business Interview specifically designed for family business takes into account intangible variables described in the organizational management literature like the systemic constructs that has a strong impact on the health of organizations. In addition to the specific contribution to management tools, it is expected that this interview may help to develop new studies and practices about organizational health in work and in family businesses.

**Disclosure of Interest:**

None Declared

